# Digital cognitive behavioral therapy for insomnia on depression and anxiety: a systematic review and meta-analysis

**DOI:** 10.1038/s41746-023-00800-3

**Published:** 2023-03-25

**Authors:** Suonaa Lee, Jae Won Oh, Kyung Mee Park, San Lee, Eun Lee

**Affiliations:** 1 Department of Psychiatry and the Institute of Behavioral Science in Medicine, Yonsei University College of Medicine, Seoul, Republic of Korea; 2 Department of Psychiatry, Yongin Severance Hospital, Yonsei University College of Medicine, Yongin, Republic of Korea; 3 Institute for Innovation in Digital Healthcare, Yonsei University, Seoul, Republic of Korea

**Keywords:** Medical research, Health care

## Abstract

Despite research into the development of digital cognitive behavioral therapy for insomnia (dCBT-I), research into the outcomes of dCBT-I on insomnia and the associated clinical conditions of depression and anxiety have been limited. The PubMed, PsycINFO (Ovid), Embase, and Cochrane databases were searched for randomized controlled trials (RCTs) on adult patients with insomnia also having reported measures of depressive or anxiety symptoms. In total, 2504 articles were identified after duplicate removal, and 22 RCTs were included in the final meta-analysis. At the post-treatment assessment, the dCBT-I group had a small to moderate effect in alleviating depressive (standardized mean difference (SMD) = −0.42; 95% CI: −0.56, −0.28; *p* < 0.001; *k* = 21) and anxiety symptoms (SMD = −0.29; 95% CI: −0.40, −0.19; *p* < 0.001; *k* = 18), but had a large effect on sleep outcome measures (SMD = −0.76; 95% CI: −0.95, −0.57; *p* < 0.001; *k* = 22). When considering treatment adherence, the treatment effects of those in the high adherent groups identified a more robust outcome, showing greater effect sizes than those in the low adherent groups for depression, anxiety, and sleep outcomes. Furthermore, additional subgroup analysis on studies that have used the fully automated dCBT-I treatment without the support of human therapists reported significant treatment effects for depression, anxiety, and sleep outcomes. The results demonstrated that digital intervention for insomnia yielded significant effects on alleviating depressive and anxiety symptoms as well as insomnia symptoms. Specifically, the study demonstrated significant effects on the above symptoms when considering treatment adherence and implementing fully automated dCBT-I.

## Introduction

Insomnia is one of the most common sleep disorders, posing a significant public health concern, with an estimated prevalence of 10–30% among adults in the general population^1,2^. These numbers are greater among patients, with reports estimating 69% prevalence among primary care patients^3^. Insomnia disorder is defined by the Diagnostic and Statistical Manual of Mental Disorders – IV (DSM-IV) as the complaint for difficulty in initiating or maintaining sleep, or restorative sleep for at least 1 month^4^. Such sleep disturbances may cause clinically significant distress or impairment in social, occupational, or other areas of functioning. Apart from fatigue, insomnia has also been associated mental disorders, low work productivity, and cognitive impairment. Despite its high prevalence and potentially severe consequences, only a limited number of people seek treatment for insomnia^5^.

Depression and anxiety are the most common comorbid mental disorders associated with insomnia which can also exacerbate the sleep disorder^6,7^. Recently, epidemiologic studies have reported that insomnia predicts the development of major depression, anxiety, and suicide. Various cross-sectional and longitudinal research have presented insomnia to be associated with an increased risk of mood and anxiety disorders as well as suicide. Those with insomnia reported increased odds of depression and anxiety as compared to those without^8^. Sleep disturbances are detected among 90% of patients with clinical depression^9^, and those with insomnia are ten times more likely to experience clinical depression^10^. As a result, insomnia can be considered a subsequent risk factor for depression due to its bidirectional relationship with depression. Likewise, insomnia is also the most prevalent sleep disturbance associated with anxiety disorders as poor sleep quality has been found among adults with anxiety disorders. In the Diagnostic and Statistical Manual for Mental disorders, fifth edition (DSM-5), sleep disturbances are one of the diagnostic criteria for generalized anxiety disorder, which is characterized by excessive anxiety and worry about certain events or activities. Moreover, studies have found generalized anxiety disorder to be the most prevalent psychiatric diagnosis among patients with insomnia, thus presenting as a significant comorbid disorder^11^.

Cognitive behavioral therapy for insomnia (CBT-I) has been an effective non-pharmacological treatment for insomnia. It is a multi-component, evidence-based treatment and is considered the first-line approach including cognitive restructuring, sleep restriction, stimulus control, sleep hygiene education, and relaxation^12,13^. Due to the association between insomnia and depression, CBT-I has been viewed an effective approach for managing depression^14^. A systematic review of CBT-I to treat depression revealed CBT-I as a promising treatment for depression comorbid with insomnia, with in-person CBT-I delivery having the most supporting evidence in its efficacy among 18 studies that included CBT-I, prescription medication or sleep hygiene as its treatment methods^14^. In addition, study findings suggest that insomnia improvement from CBT-I may also mediate the reduction in depressive symptoms. Likewise, in addition to depression, CBT-I demonstrated moderate to large effect sizes for generalized anxiety disorder symptoms^15^. These findings indicate that CBT-I is not only effective for treating insomnia and sleep-related disorders but also for treating comorbid mental disorders including depression and anxiety.

Whilst such a traditional approach of CBT-I has been proven effective, there are certain limitations of this therapeutic method including the lack of therapists, time and geographical limitations, and high costs. With the advent of technology, digital CBT-I (dCBT-I), which is the implementation of technology in computers, the internet, smartphone applications, and other devices in healthcare service have been developed and researched over the last decade^13,16^. dCBT-I programs are not only structured with the main key components of CBT-I but also provide additional levels of personalized support to enhance user engagement, including the use of email reminders, alerts, etc. Furthermore, dCBT-I users can evaluate their sleep status through online sleep diaries, questionnaires, or syncing with other devices such as wrist-worn actigraphs, to track certain sleep patterns and collect the ecological momentary assessment. A meta-analysis found that internet-based CBT-I had significantly improved insomnia severity and sleep parameters in addition to comorbid factors of depression and anxiety, maintaining such improvements at a 6-month follow-up. According to these results, dCBT-I is an effective treatment alternative for insomnia, both in terms of clinical effectiveness and positive user satisfaction whilst also demonstrating that the treatment was effective in improving comorbid anxiety and depression with a mild strength^12^.

However, despite the research on the effects of dCBT-I, further investigations are needed to evaluate the outcomes of dCBT-I on insomnia and the comorbid factors depression and anxiety, as only a small handful of studies were included in the previous meta-analysis^17^. Furthermore, the implications of the treatment adherence and the effects of therapist’s involvement when using such treatment methods has received relatively little attention^18^. Thus, the current meta-analysis aimed to assess the effects of dCBT-I on depression and anxiety symptoms as well as insomnia and other sleep parameters, including total sleep time (TST), sleep efficiency (SE), sleep onset latency (SOL), and wake after sleep onset (WASO) by pooling published randomized control trials (RCTs). This would assist in determining the efficacy of dCBT-I on insomnia as well as the most typical comorbid factors, depression and anxiety, with in consideration of adherence rates and in-person involvement of the therapists. Furthermore, adherence rates must be taken into account to establish whether an outcome is related to a certain treatment^19^. Whilst there are various studies that investigate mobile health (mHealth) devices supporting patients and healthcare systems for medication adherence^20,21^, a robust definition of adherence rate in actual treatment methods delivered using mHealth technology is currently absent. A review of mHealth technology identified adherence can be measured in various methods such as the number of logins, completed modules, pages viewed and completed self-reported measures^22^. Others also suggested the usage time of these devices^23^. Following these prior studies and their definitions of mHealth technology adherence, we have defined adherence based on the percentage of participants who had fully completed the provided dCBT-I sessions.

## Results

### Study flow

**Fig. 1:**
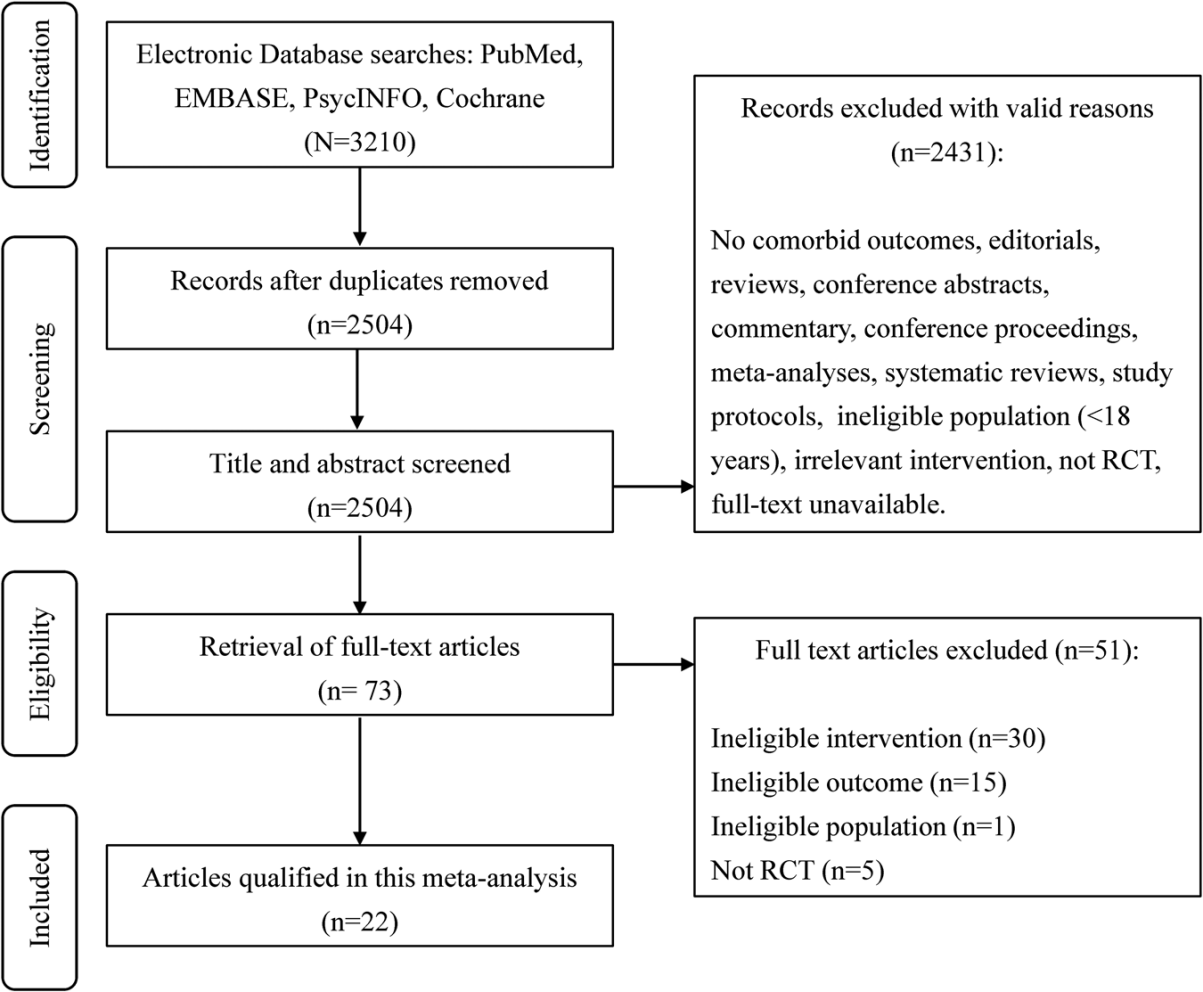
PRISMA flow diagram. Search and study selection process.

The flow of study selection is presented in the PRISMA flow diagram (Fig. 1). A total of 2504 articles were identified after duplicate removal, of which, 73 articles were assessed for full-text review. A final sample of 22 RCTs was included in the meta-analysis.

### Study characteristics

The characteristics of the 22 studies included in the meta-analysis are described in Table 1. The meta-analysis included a total of 10,486 participants, of whom 5494 were randomized to the dCBT-I group, with a median study size of 111 participants (range 21–3755 participants). The overall mean age of dCBT-I and control groups was 43.8 ± 8.7 years and 43.6 ± 8.3 years, respectively. Participants in control conditions received active interventions including sleep education or general health education (not specifically targeting sleep), or passive controls including treatment as usual and wait-lists. All studies used a parallel design, with 20 studies using two-arm trials and two studies using three-arm trials. For treatment groups, the dCBT-I therapy sessions ranged between 5 and 8 sessions with an average of 6.18 sessions across the included studies. The studies were mostly conducted in Europe (*n* = 13) and the United States (*n* = 5). Baseline mean depression scores from measurements indicated 11 studies included participants with subthreshold symptoms of depression, 1 study included participants with clinically significant depression symptoms, and 9 studies with participants having no depression symptoms. Baseline mean anxiety scores from measurements indicated 9 studies included participants with subthreshold symptoms of anxiety and 7 studies with no anxiety symptoms. One study used anxiety measurement that did not provide a cut-off score for interpretation^24^. By providing only mean difference in anxiety, it was difficult to obtain baseline mean anxiety scores in the study conducted by Glozier et al^25^. Furthermore, among the 22 studies included, 12 reported completion rates for dCBT-I sessions, with the average completion rate of 59.73%.

**Table 1:** Summary characteristics of the included studies.

Author	Year	Country	Total sample size (%Female)	Mean age, years (SD)	Study design	dCBTi components	No. of sessions	Time point of post-assessment	Insomnia outcome measurement	Depression outcome measurement	Anxiety outcome measurement
Agyemang et al^48^.	2017	United States	28 (78.6)	dCBTi: 43 (10.2) TAU: 50 (10.4)	2 parallel arms (dCBTi, TAU)	SRT, SC, CR, SHE	6	9–10 weeks	ISI, TST, SE, SOL WASO	PHQ-9	GAD-7
Ahorsu et al^49^.	2020	Iran	320 (58.4)	dCBTi: 38.37 (13.45) Sleep education: 37.99 (9.88)	2 parallel arms (dCBTi, sleep education)	SHE, RE, CR, SRT, SC	6	10 weeks	ISI, TST, SE, WASO, SOL, PSQI	HADS	HADS
Blom et al^50^.	2015	Sweden	48 (47.9)	dCBTi: 56.1 (10.2) GCBT: 52.6 (16.6)	2 parallel arms (dCBTi, GCBT)	SRT, SC, SHE, CR, RE, RP	8	8 weeks	ISI, TST SE, SOL	MADRS-S	–
Bostock et al^51^.	2016	United Kingdom	270 (33.3)	dCBTi: 33.9 (6.41) WL: 33.3 (5.59)	2 parallel arms (dCBTi, WL)	SRT, SC, CR, RE, SHE	6	8 weeks	SCI	PHQ-2	GAD-2
Cheng et al^52^.	2018	United States	1385 (78.9)	dCBTi: 44.5 (15.8) Online sleep education: 45.7 (15.1)	2 parallel arms (dCBTi, online sleep education)	SRT, SC, CR, PI, RE, SHE	6	12 weeks	ISI	QIDS	–
Christensen et al^53^.	2016	Australia	1149 (73.5)	dCBTi: 42.95 (12.17) Online attention-matched placebo: 42.51 (12.24)	2 parallel arms (dCBTi, online attention-matched placebo)	SRT, SC, CR, SHE, RP	6	6 weeks	ISI	PHQ-9	GAD-7
Espie et al^54^.	2019	United Kingdom	1711 (77.7)	dCBTi: 48.4 (13.9) SHE: 47.7 (13.6)	2 parallel arms (dCBTi, SHE)	SRT, SC, RE, CR, PI, PE, SHE	6	8 weeks	SCI	PHQ-9	GAD-7
Freeman et al^55^.	2017	United Kingdom	3755 (71.3)	dCBTi: 24.8 (7.7) TAU: 24.6 (7.6)	2 parallel arms (dCBTi, TAU)	SRT, SC, RE, CR, PI, SHE, MI, IM	6	10 weeks	ISI	PHQ-9	GAD-7
Felder et al^56^.	2020	United States	208 (100)	dCBTi: 33.90 (3.38) TAU: 33.2 (4.0)	2 parallel arms (dCBTi, TAU)	SRT, SC, CR, RE, SHE	6	10 weeks	ISI, PSQI, SE	EPDS	GAD-7
Glozier et al^35^.	2019	Australia	87 (0)	dCBTi: 58.6 (6.3) Online PE: 58.1 (6.1)	2 parallel arms (dCBTi, Online PE)	SRT, SC, SHE, CR, RP	6	12 weeks	ISI	CES-D	STPI
Kalmbach et al^57^.	2020	United States	91 (100)	dCBTi: 28.91 (28.91) digital sleep education: 29.16 (4.11)	2 parallel arms (dCBTi, digital sleep education)	SRT, SC, CR, PI, RE, SHE	6	7 weeks	ISI, PSQI	EPDS	–
Krieger et al^58^.	2019	Switzerland	104 (68.3)	dCBTi: 42.17 (12.4) SRT: 46.59 (17.52) TAU: 45.24 (12.40)	3 parallel arms (MCT + TAU, SRT + TAU, TAU)	PE, SRT, RE, CR, SHE, RP	8	8 weeks	ISI, PSQI, SE	ADS-K	–
Kyle et al^59^.	2020	United Kingdom	410 (86.6)	dCBTi: 52.5 (11.2) WL: 52.4 (11.7)	2 parallel arms (dCBTi, WL)	SRT, SC, CR, SHE, RE	6	10 weeks	ISI, PSQI	PHQ-9	GAD-2
Lancee et al^60^.	2015	Netherlands	63 (79.4)	dCBTi: 47.47 (14.37) WL: 49.98 (13.71)	2 parallel arms (dCBTi, WL)	PE, RE, SRT, SHE, CR	6	12 weeks	ISI, TST, SE, WASO, SOL	CES-D	HADS
Lancee et al^61^.	2016	Netherlands	90 (81.1)	dCBTi: 41.2 (14.1) ftf: 38.5 (13.1) WL: 45.1 (13.7)	3 parallel arms (dCBTi, ftf, WL)	PE, RE, SHE, SRT, CR	6	12 weeks	ISI, TST, SE	CES-D	HADS
Lorenz et al^34^.	2019	Switzerland	56 (69.6)	dCBTi: 41.72 (17.31) WL: 44.04 (20.05)	2 parallel arms (dCBTi, WL)	PE, SRT, SC, RE, SHE, RP, CR	6	6 weeks	ISI, TST, SE, WASO, SOL	BDI-II	BSI-Anxiety
Majd et al^62^.	2020	Iran	312 (55.8)	dCBTi: 36.21 (5.81) Sleep education: 35.29 (5.76)	2 parallel arms (dCBTi, sleep education)	SHE, SC, RE, CR, SRT, PI	6	10 weeks	ISI	HADS	HADS
Pillai et al^63^.	2015	United States	21 (62.5)	dCBTi: 53.2 (12.2) Sleep education: 44.0 (13.2)	2 parallel arms (dCBTi, sleep education)	SRT, SC, CR, PI, RE, SHE	6	7 weeks	ISI, TST, SOL	–	BAI
Sveen et al^64^.	2021	Sweden	21 (66.7)	dCBTi: 49.9 (5.8) Sleep education: 45.6 (5.5)	2 parallel arms (dCBTi, sleep education)	SRT, SC, ST, CR, SHE	8	9 weeks	ISI	MADRS	GAD-7
van der Zweerde et al^65^.	2019	Netherlands	104 (81.7)	dCBTi: 44.64 (13.12) SM: 46.29 (15.07)	2 parallel arms (dCBTi, SM)	SHE, SR, SC, RE, CR, RP	5	9 weeks	ISI, TST, SE, WASO, SOL	PHQ-9	HADS
van der Zweerde et al^66^.	2020	Netherlands	134 (64.9)	dCBTi: 51.7 (15.77) TAU: 49.4 (16.01)	2 parallel arms (dCBTi, TAU)	SHE, SRT, SC, CR, RP, RE	5	8 weeks	ISI, TST, SE, SOL, WASO	HADS	HADS
van Straten et al^67^.	2014	Netherlands	118 (70.3)	dCBTi: 48.7 (13.8) WL: 50.1 (11.9)	2 parallel arms (dCBTi, WL)	PE, SHE, SRT, SC, RE, CR	6	6 weeks	PSQI, TST, SOL, SE	CES-D	HADS

*ADS-K* Allgemeine Depressions-Skala-Kruzform, the German short version of the Center for Epidemiological Studies Depression Scale, *BAI* Beck Anxiety Inventory, *BDI-II* Beck depression inventory, second edition, *BSI-A* Anxiety subscale of the Brief symptom inventory, *CES-D* Center for Epidemiological Studies Depression Scale, *CR* Cognitive restructuring, *EPDS* Edinburgh Postnatal Depression Scale, *ftf* face-to-face, *GAD* Generalized Anxiety Disorder, *GCBT* Group Cognitive behavioral therapy, *GSII* Glasgow Sleep Impact Index, *HADS* Hospital Anxiety and Depression Scale, *HAM-D* Hamilton Depression Rating Scale, *ISI* Insomnia severity index, *IM* Imaginary, *MCT* Multicomponent internet-based guided treatment, *MADRS* Montgomery Åsberg Depression Rating Scale, *MADRS-S* Montgomery Åsberg Depression Rating Scale-Self rated, *MI* Mindfulness and/or mediation, *PE* Psychoeducation, *PHQ* Patient Health Questionnaire, *PI* Paradoxical intention, *PSQI* Pittsburgh Sleep Quality Index, *QIDS* Quick Inventory of Depression Symptomology – Self Report, *RE* Relaxation techniques, such as progressive muscle relaxation, *RP* Relapse prevention, *SC* Stimulus control, *SCI* Sleep Condition Indicator, *SE* Sleep efficiency, *SHE* sleep hygiene education, *SM* sleep diary monitoring, *ST* Stress management, *STPI* State Trait Personality Inventory, *SRT* sleep restriction therapy, *TAU* treatment as usual, *WL* wait-list

### Risk of bias

The risks of bias for the included studies were assessed using the Cochrane Risk of Bias tool – version 2^26^ and the results are presented in Supplementary Fig. 1. The overall risk of bias was low for 8 studies, moderate for 10, and high for the remaining 4 studies across five domains. A significant risk of bias was detected from the measurement of the outcome domain, predominantly due to studies utilizing self-rating questionnaires as their primary outcomes. Furthermore, blinding of participants and research personnel may have also contributed to deviations from the intended intervention.

### Treatment effects

#### Depression measures

**Fig. 2:**
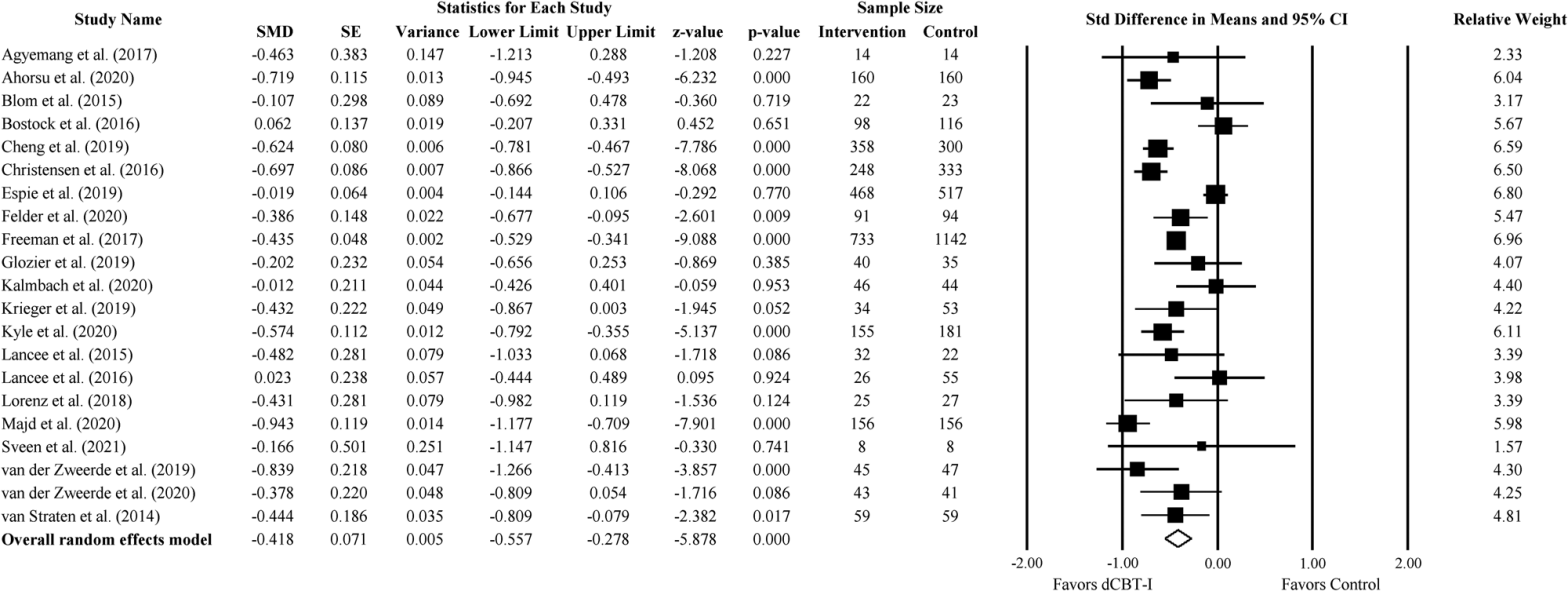
The effect of dCBT-I on depression. Forest plot of studies reporting the effect of dCBT-I on depression.

Twenty-one out of 22 studies reported the severity of depressive symptoms. The outcome measures of depressive symptoms varied across studies including Center for Epidemiologic Studies Depression Scale (CES-D), Patient Health Questionnaire-9 (PHQ-9), Patient Health Questionnaire-2 (PHQ-2), Edinburgh Postnatal Depression Scale (EPDS), Allgemeiner Depressions-Skala (ADS-K), Montgomery–Åsberg Depression Rating Scale (MADRS), Hospital Anxiety and Depression Scale-Depression (HADS-D), Beck Depression Inventory (BDI-II), and Quick Inventory of Depressive Symptomatology (QIDS). At the post-treatment assessment, we found a small to moderate effect favoring dCBT-I (Fig. 2; Standardized Mean differences (SMD) = −0.42; 95% confidence interval (CI): −0.56, −0.28; *p* < 0.001; *k* = 21). The statistical heterogeneity in effect sizes among studies was high (*I*^*2*^ = 81.79; *Q* = 109.85; df = 20; *p* < 0.001).

#### Anxiety measures

**Fig. 3:**
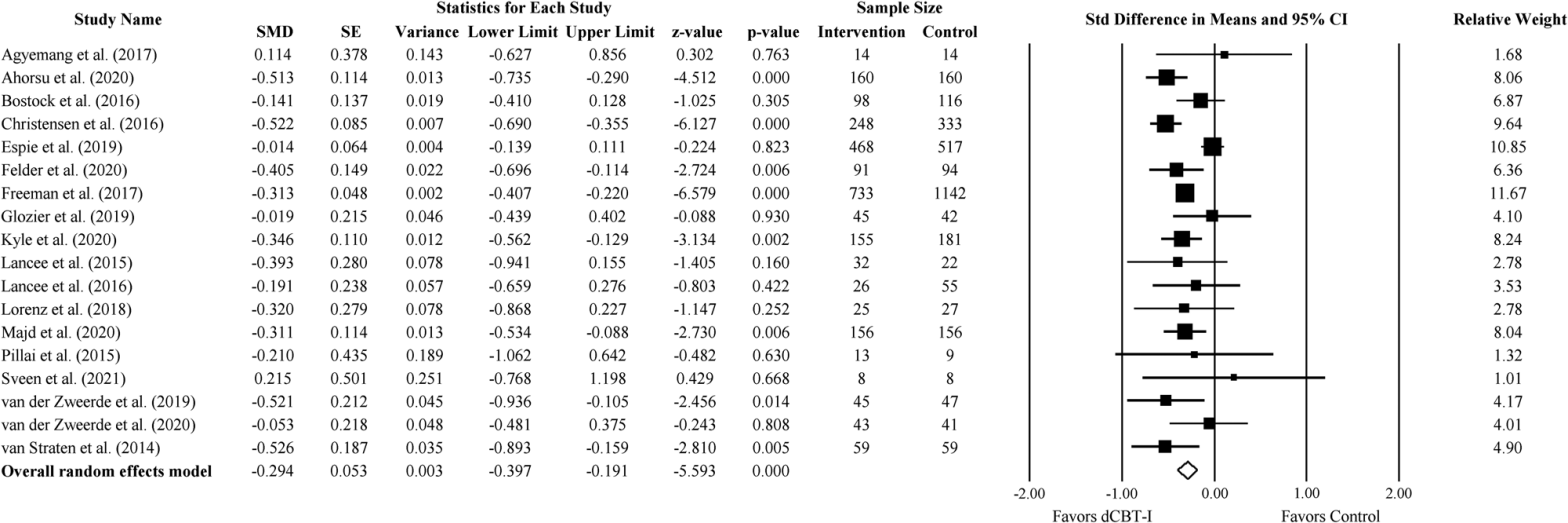
The effect of dCBT-I on anxiety. Forest plot of studies reporting the effect of dCBT-I on anxiety.

Eighteen out of 22 studies reported the severity of anxiety symptoms. The outcome measures of anxiety symptoms varied across studies including General Anxiety Disorder-7 (GAD-7), General Anxiety Disorder-2 (GAD-2), Hospital Anxiety and Depression Scale-Anxiety (HADS-A), Brief Symptom Inventory-Anxiety (BSI-Anxiety), and Beck Anxiety Inventory (BAI). For anxiety symptoms at the post-treatment assessment, we found a small to moderate effect favoring dCBT-I (Fig. 3; SMD = −0.29; 95% CI: −0.40, −0.19; *p* < 0.001; *k* = 18). The statistical heterogeneity in effect sizes among studies was high (*I*^*2*^ = 57.75; *Q* = 40.24; df = 17; *p* < 0.001).

#### Sleep measures

**Fig. 4:**
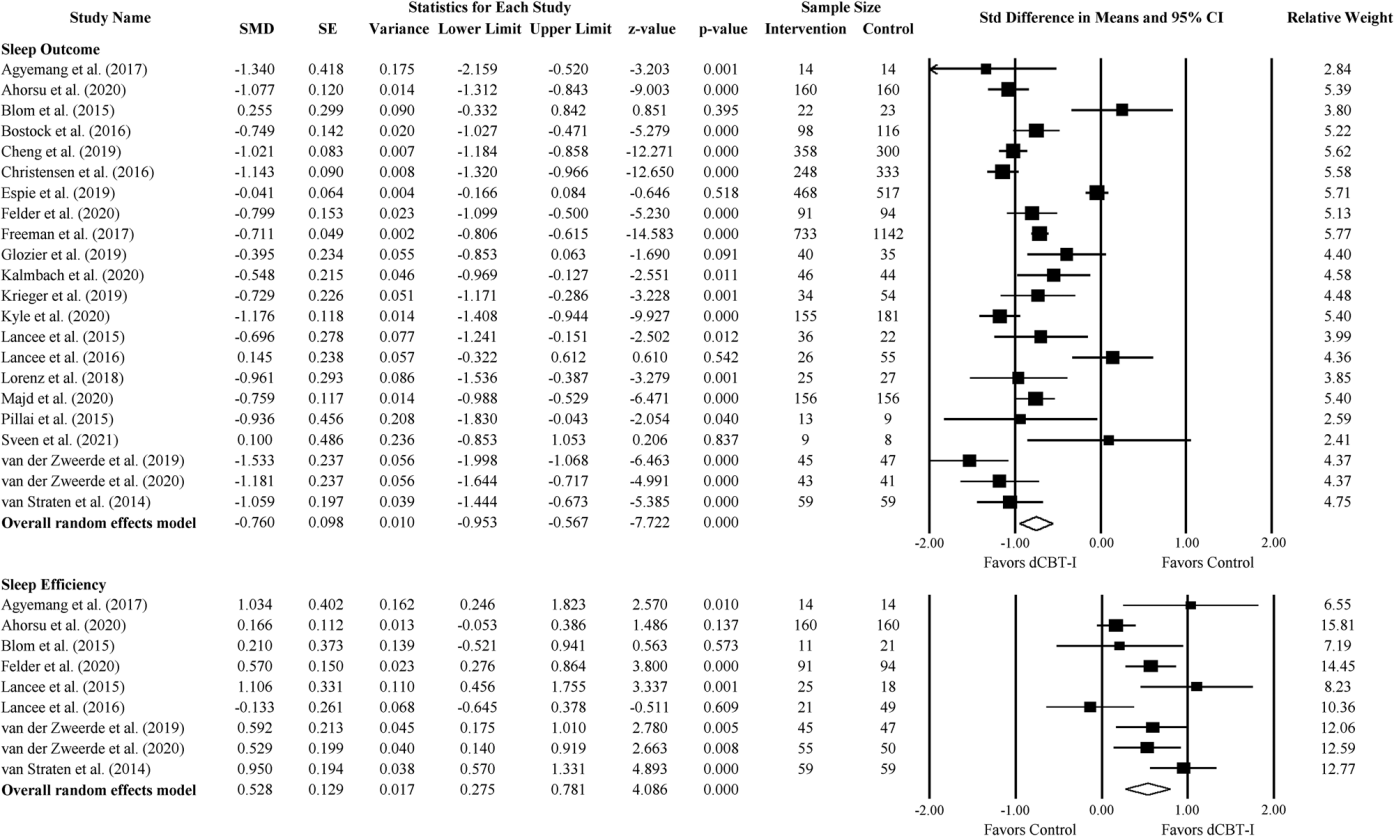
The effect of dCBT-I on sleep outcome and sleep efficiency. Forest plot of studies reporting the effect of dCBT-I on sleep outcome and sleep efficiency.

The sleep outcome measures varied across studies and included Insomnia Severity Index (ISI), Sleep Condition Indicator (SCI), and Pittsburgh Sleep Quality Index (PSQI). Where available, ISI was chosen for the main sleep outcome measurement. All but three studies used ISI as an outcome for insomnia severity. These studies were Bostock et al. (2016), Espie et al. (2019) and van Straten et al. (2014). Among the three remaining studies, two studies used the SCI, while one study used the PSQI measure. For the severity of insomnia post-treatment, we found a large effect favoring dCBT-I (Fig. 4; SMD = −0.76; 95% CI: −0.95, −0.57; *p* < 0.001; *k* = 22). The statistical heterogeneity in effect sizes among studies was high (*I*^*2*^ = 90.59; *Q* = 223.04; df = 21; *p* < 0.001). In studies including only ISI, we found a large effect favoring dCBT-I (Supplementary Fig. 3; SMD = −0.81; 95% CI: −0.97, −0.65; *p* < 0.001; *I*^*2*^ = 79.51; *k* = 19).

For sleep diary outcomes, the effect was significant with a moderate to large effect size for SE (Fig. 4; SMD = 0.53; 95% CI: 0.28, 0.78; *p* < 0.001; *I*^*2*^ = 68.91; *k* = 9), SOL (Supplementary Fig. 2; SMD = −0.65; 95% CI: −1.14, −0.15; *p* = 0.01; *I*^*2*^ = 89.00; *k* = 8) and WASO (Supplementary Fig. 2; SMD = −1.48; 95% CI: −2.76, −0.20; *p* = 0.03; *I*^*2*^ = 97.17; *k* = 5), while the effect was significant with a small effect size for TST (Supplementary Fig. 2; SMD = 0.26; 95% CI: 0.04, 0.50; *p* = 0.02; *I*^*2*^ = 53.03; *k* = 9).

### Sensitivity analysis

Sensitivity analysis was conducted by removing two studies including participants with medical comorbidities (cancer and epilepsy) and one study with major depressive disorder. After excluding the studies, robust treatment effects of dCBT-I were demonstrated for depression (Supplementary Fig. 4; SMD = −0.41; 95% CI: −0.56, −0.25; *p* < 0.001; *I*^*2*^ = 83.36; *k* = 18), anxiety (Supplementary Fig. 5; SMD = −0.30; 95% CI: −0.40, −0.19; *p* < 0.001; *I*^*2*^ = 58.17; *k* = 15) and sleep (Supplementary Fig. 6; SMD = −0.74; 95% CI: −0.95, −0.53; *p* < 0.001; *I*^*2*^ = 91.40; *k* = 19) outcomes. When sensitivity analysis was performed after excluding 4 studies with high risk of bias, the treatment effects were robust for depression (SMD = −0.40; 95% CI: −0.55, −0.25; *p* < 0.001; *I*^2^ = 83.84; *k* = 18), anxiety (SMD = −0.29; 95% CI: −0.41, −0.18; *p* = 0.001; *I*^2^ = 65.13; *k* = 14), and sleep outcomes (SMD = −0.74; 95% CI: −0.95, −0.53; *p* < 0.001; *I*^2^ = 91.86; *k* = 18).

### Subgroup analysis

#### Treatment adherence

**Fig. 5:**
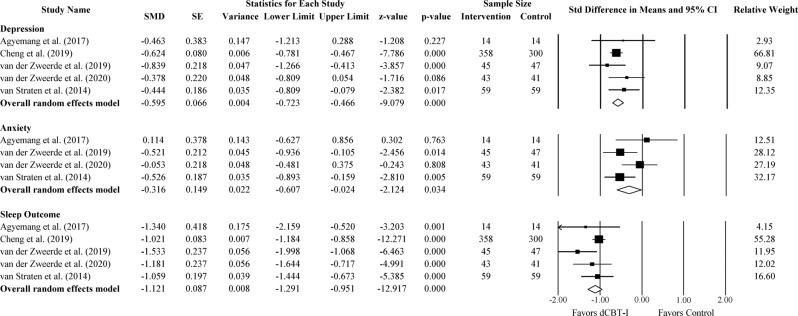
The effect of dCBT-I among dCBT-I completers (>65%). Forest plot of studies reporting the effect of dCBT-I among dCBT-I completers with more than 65% of completion rate.

Additional subgroup analysis was performed on 12 studies that reported the number of participants who completed the dCBT-I sessions. To compare the effect of treatment adherence, we divided 12 studies into two groups: (1) high adherent group with >65% of dCBT-I completers; (2) low adherent group with <65% of dCBT-I completers. The treatment effects of the high adherent group were significant for depression (SMD = −0.60; 95% CI: −0.72, −0.47; *p* < 0.001; *I*^*2*^ = 0.00; *k* = 5), anxiety (SMD = −0.32; 95% CI: −0.61, −0.02; *p* = 0.03; *I*^*2*^ = 38.58; *k* = 4) and sleep outcomes (SMD = −1.12; 95% CI: −1.30, −0.95; *p* < 0.001; *I*^*2*^ = 15.17; *k* = 5). See Fig. 5 for the detailed results of this analysis. For the low adherent group, the treatment effects were also significant but effect sizes were smaller than those in adherent groups for depression (SMD = −0.35; 95% CI: −0.57, −0.14; *p* = 0.001; *I*^*2*^ = 88.71; *k* = 7), anxiety (SMD = −0.28; 95% CI: −0.45, −0.11; *p* = 0.001; *I*^*2*^ = 82.34; *k* = 6), and sleep outcomes (SMD = −0.69; 95% CI: −1.05, −0.34; *p* < 0.001; *I*^*2*^ = 95.82; *k* = 7).

#### Effects of fully automated dCBT-I

The additional subgroup analysis was performed on 14 studies using fully automated dCBT-I without support of human therapists. The treatment effects of the fully automated dCBT-I were significant for depression (SMD = −0.43; 95% CI: −0.61, −0.26; *p* < 0.001; *I*^2^ = 88.14; *k* = 13), anxiety (SMD = −0.29; 95% CI: −0.41, −0.17; *p* = 0.001; *I*^2^ = 68.46; *k* = 12), and sleep outcomes (SMD = −0.81; 95% CI: −1.04, −0.59; *p* < 0.001; *I*^2^ = 92.69; *k* = 14). The detailed results of this analysis are presented in Supplementary Fig. 7.

### Publication bias

Visual inspection of funnel plots, (Supplementary Fig. 8 for sleep outcome, Supplementary Fig. 9 for depression, and Supplementary Fig. 10 for anxiety) and Egger’s tests for asymmetry in funnel plots were used to estimate publication bias. The Egger’s tests were not significant for depression (*t* = 0.03, df = 19, *p* = 0.98), anxiety (*t* = 0.02, df = 16, *p* = 0.98), and insomnia (*t* = 0.63, df = 20, *p* = 0.54), indicating no significant publication bias.

## Discussion

The current meta-analysis aimed to assess the efficacy of dCBT-I and examine the impact of adherence to dCBT-I on treatment outcomes of depressive and anxiety symptoms, and sleep disorders. By pooling the data obtained from eligible RCTs, our results demonstrated that digital intervention for insomnia yielded significant effects at post-treatment as compared to control conditions on alleviating depressive and anxiety symptoms as well as insomnia symptoms, SE, TST, SOL, and WASO.

The results were comparable to the findings reported in the previous meta-analysis examining the effects of dCBT-I on depression and anxiety, which showed small to moderate effects on depression and anxiety^27^. However, the previous study was limited by the relatively small number of available studies (10 RCTs). With a substantially larger sample size of 22 RCTs, this updated meta-analysis further supported the efficacy of dCBT-I. Furthermore, we extended the findings of the meta-analysis conducted by Ye et al., by demonstrating that fully automated dCBT-I interventions without the support of human therapists, are also effective for improving conditions of depression and anxiety^12^. One previous study demonstrated a fully automated dCBT-I integrated into an existing UK-based clinical service, demonstrating its effectiveness in alleviating depression, anxiety, and insomnia^28^. Given that published trials on the automated dCBT-I implementations in real-world environments are scarce, the potential effects of a fully automated version of dCBT-I for people with depression or anxiety warrant further evaluation.

Although the pooled effect of dCBT-I on depressive and anxiety symptoms is small to moderate, there was considerable heterogeneity in the magnitude of the effects observed. This heterogeneity is comparable to previous research^29^ and expected given the diversity of participants recruited, outcome measures, the delivery format of CBT-I, and baseline severity levels of depression and anxiety in the included studies. The effects of dCBT-I interventions on depression and anxiety symptoms were relatively robust after removing the three studies that included participants with mental or medical comorbidities. Considering that the majority of the studies included in this meta-analysis had subclinical depression and anxiety samples, this suggests that dCBT-I interventions are beneficial in reducing subclinical depression and anxiety symptoms. Whilst dCBT-I is developed for insomnia treatment, current findings suggest that dCBT-I has the capability for an effective supplementary therapy beyond its current potential.

Apart from the mitigation of depression and anxiety symptoms, the improvement in insomnia severity in this study is generally well in line with those reported in a previous meta-analytic review of dCBT-I for insomnia severity, SE, TST, SOL, and WASO^13^. Nonetheless, a direct comparison with the meta-analysis conducted by Soh et al. is difficult as they calculated the effect size as mean differences^13^. This shows dCBT-I as an effective treatment method, not just as an adjunct to pharmacological or psychotherapeutic treatment for depression and anxiety.

Prior research has reported treatment adherence to be positively associated with treatment effectiveness of technology-mediated treatments^30^. As an extension of this, our study investigated the effects of dCBT-I adherence on depression, anxiety, and insomnia outcomes by considering the proportion of the participants who completed all dCBT-I sessions. The effect sizes for depression, anxiety, and insomnia severity were comparatively greater in high adherent group although the treatment effects were significant in low adherent groups as well. This presents the adherence moderates the effect of the dCBT-I intervention.

Nonetheless, previous research has identified that even the most effective apps have minimal effect if these lack user engagement, resulting in a high attrition rate^31^. The attrition-efficacy gap needs to be settled especially for those requiring sustained mental health treatment^32^. The problem of high dropout rates is especially true for fully automated dCBT-I intervention without any support of human therapists^32,33^. Therefore, adherence-promoting features such as ease of use, rewards, ability to personalize app, tailored interventions, social or peer support in app, personalized feedback, and integration with clinical services should be considered^34^. Although there’s lacking evidence in research comparing the differences between automated support and with or without human support, automated reminders have increased enhanced adherence to treatment^35^. The fears around security and privacy inherent to digital interventions might be an additional factor in adherence and attrition for some participants, therefore user safety should be considered upfront^32^. Furthermore, most studies showed various methods to assess adherence, which make it difficult to compare outcomes meaningfully, though adherence was most often assessed by the degree of program completion^36^. Therefore, a standardized method for assessing adherence is required to reliably predict the impacts of adherence on treatment outcomes.

Given that few of the studies included in the current review involve participants with clinically significant level of depression and anxiety symptoms, our result of significant effects favoring dCBT-I could be seen as pertaining to patients with subthreshold level of depression and anxiety symptoms. In a previous study of internet-delivered CBT-I, when comparing the differences between severe and low to mildly depressed patients, those with severe symptoms more likely to benefit from human support of reminding and encouraging patients by e-mail, while those with low level of depressive symptoms were demonstrated to benefit adequately regardless of the support^37^. This indicates that the addition of some guidance could be preferred depending on the baseline severity of depression although fully automated intervention increases scalability. Thus, further research is needed to determine the role of symptom severity of depression and anxiety for the effect of digital intervention.

This meta-analysis supports the efficacy of dCBT-I on insomnia and subclinical symptoms of depression and anxiety symptoms. The current study demonstrated small-to-moderate effect sizes, which was consistent with prior meta-analyses conducted to evaluate the effectiveness of cognitive behavior therapies for depression, anxiety, and sleep disorders^38^. Studies have identified mostly small to moderate effect when having treatment as usual or pill placebo as the control condition^39^. Henceforth, based on these results the small-to-moderate effects of dCBT-I treatment can be considered as a clinically meaningful outcome. This study also demonstrated that fully automated dCBT-I interventions were able to alleviate comorbid depression and anxiety symptoms with insomnia. To the best of our knowledge, there haven’t been earlier studies conducting meta-analysis to investigate the treatment effect of fully automated dCBT-I. Overall, the results demonstrate greater effect sizes for patients utilizing fully automated dCBT-I, in addition to the significant effects of treatment adherence.

This meta-analysis had some limitations. First, 12 out of 22 studies had a small sample size of <50 which could lead to an overestimation of effect sizes. Second, due to the heterogeneity in the details reported, long-term outcomes were difficult to evaluate between studies. Also, details regarding the baseline severity of depression and anxiety of the participants were also not clearly presented; therefore, it was difficult to identify differences between the studies included. Further studies especially inclusive of individuals with clinical depression or anxiety should be explored. Finally, the control groups were not consistent among the included studies comparing the dCBT-I group intervention with the waitlist, treatment as usual, and psychoeducation, implicating the heterogeneity of the analyses. To explore and determine the effectiveness of dCBT-I, future research should first consider having a consistent control group in addition to potentially comparing the dCBT-I with individual face-to-face CBT-I interventions. Furthermore, whilst the current study did not investigate the interaction effects between adherence levels with the type of dCBT-I delivery, whether or not the treatment delivery was fully automated, future studies may consider this interaction effect in their research. The outcomes may show a clinically meaningful interpretation regarding adherence levels and the different types of dCBT-I treatment delivery.

The results of our meta-analysis emphasize the need for CBT-I by digital means in patients with depression and anxiety symptoms. Since dCBT-I can be implemented globally, further research is needed to provide sufficient clinical evidence of its effectiveness, especially in the fully automated version in comparison to the traditional methods of face-to-face CBT-I.

## Methods

### Data sources and searches

This study was conducted in reference to the Cochrane Handbook for Systematic Reviews of Interventions^40^ and reported according to the Preferred Reporting for Items for Systematic Reviews and Meta-Analyses (PRISMA) guidelines^41^. The review protocol was prospectively registered on the International Prospective Register of Systematic Reviews (PROSPERO), registration number: CRD42022315203. There was no prior published protocol for the current study. Furthermore, PubMed, Embase, PsycINFO, and Cochrane databases were accessed to search for studies published from inception to January 15th, 2022. Full search strategies are attached in Supplementary Table 1.

### Study selection

The following inclusion criteria were established for study selection: (1) comprised adult patients aged ≥18 years; (2) have been formally diagnosed or had self-reported symptoms of insomnia defined by any edition of the DSM^42^, International Classification of Sleep Disorders^43^, or International Classification of Diseases;^44^ (3) with reported measures of depressive or anxiety symptoms; (4) involving a dCBT-I intervention delivered by digital technology including computer, Internet and smartphone applications, alongside a control group with other interventions for managing insomnia, active controls, waitlist, or participants who underwent usual care; (5) adopted an RCT design. In the current study, dCBT-I consisted of multimodal components with at least one key cognitive strategy (cognitive restructuring) and one key behavioral strategy (stimulus control or sleep restriction). Henceforth, only CBT-I methods were considered as treatment methods for this study. Furthermore, as long as the main CBT-I treatment methods were delivered via a digital device listed above, studies with additional feedback interactions via online guidance, emails and text messages were also considered to meet the selection criteria. The two researchers (SAL, JWO) independently extracted and reviewed the studies to consider their inclusion based on the eligibility criteria. Duplicate articles were removed; titles and abstracts were screened for study inclusion. Full texts of the remaining studies were further reviewed. The two reviewers (SAL, JWO) assessed inter-rater reliability using Cohen’s Kappa value, keeping the researchers blinded to each other’s decisions throughout the review process. All authors discussed any disagreement between studies and reached a consensus. The inter-rater reliability of study selection was considered strong (Kappa = 0.82).

### Data extraction and risk of bias assessment

Two authors (SAL, JWO) each extracted data from the included studies. Details including title, authors, year of publication, study design, number of dCBT-I sessions, and treatment duration were extracted in addition to sample size, mean age of each intervention, and control groups. Moreover, assessment tools were used to evaluate the relevant study variables, and pre and post-scores of both intervention and control groups were extracted. Any discrepancies were resolved through discussion among all authors.

The revised Cochrane risk of bias tool for randomized trials was used by the two researchers, independently assessing the risk of bias of each included study. Five different domains were assessed: (1) randomization process; (2) deviations from intended interventions; (3) missing outcome data; (4) measurement of the outcome; and (5) selection of the reported result. The risk of bias was assessed and reported as “low risk,” “some concerns,” or “high risk of bias.” Again, any discrepancies in the results were discussed to reach a consensus.

### Treatment outcomes

Self-reported insomnia-related measures including ISI, SCI, and PSQI were evaluated in addition to various sleep diary outcomes such as TST, SE, SOL, and WASO. Symptoms of depression were measured using the CES-D, PHQ-9, PHQ-2, EPDS, ADS-K, MADRS, HADS-D, and BDI-II. Whereas, GAD-7, GAD-2, HADS-A, BSI-Anxiety, and BAI were used to assess anxiety. These outcome measures were used to determine the efficacy of dCBT-I delivery approaches. Any missing information from the included studies was obtained by contacting the original study authors via email.

### Data synthesis and analysis

Statistical analyses were performed using Comprehensive Meta-Analysis software (version 3; Biostat Inc., Englewood, New Jersey, USA). SMDs with 95% CIs were reported for sleep diary measures, insomnia, depression, and anxiety symptoms. The overall between-group SMDs were calculated based on the differences in the post-intervention outcome measures between the dCBT-I intervention and control groups. Changes between baseline and post-intervention were not evaluated. This is in accordance with previous studies which have conducted analysis on between-group comparison of post treatment values^29^. Studies have demonstrated analyzing SMDs of post scores only is less prone to bias in comparison to utilizing the change value between baseline and post means, hence advising to avoid pre-post effect sizes in meta-analyses^45,46^. A pairwise meta-analysis was performed using the Der-Simonian and Laird random-effects model to compare the treatment effect differences. Heterogeneity was assessed using the Cochrane Q test with a statistical significance of *P* < 0.05 and *I*^2^ statistics. Egger’s test was also used to assess the potential publication bias.

Furthermore, subgroup analysis for the adherence of participants was performed. As per the aim of the study, adherence in this analysis was defined as the percentage of dCBT-I treatment module completers in each study included. Studies were divided into those with high adherence in comparison to those with low. Considering the variation of the participants’ treatment completion between the studies, a threshold value was determined based on prior research into adherence to insomnia treatment. A meta-analysis on the technology-mediated insomnia treatments found user adherence reported in various forms, including self-report measures and treatment program completion based on user login frequency recordings^30^. Approximately 41% of the participants met the adherence criteria based on the submitted response of self-report assessments, whilst user logs found 64% of participants completed the required sessions. From the two measures, an average of 52% of insomnia patients had completed their treatments and relevant self-report assessments. Another systematic review into the adherence of cognitive behavioral therapy for insomnia reported a mean adherence rate of 65.5%^47^. Thus, in line with these studies, the threshold value for the current meta-analysis was set at 65%, whereby studies with more than 65% participant who have completed the provided dCBT-I programs were determined as high treatment adherent studies.

### Reporting summary

Further information on research design is available in the Nature Research Reporting Summary linked to this article.

## Supplementary information

Supplementary Materials

REPORTING SUMMARY

## Data Availability

Data collected and used in this meta-analysis can be requested from the corresponding author.

## Code availability

No custom code or mathematical algorithms were used this study.
