# Supplementary material for: Digital cognitive behavioral therapy for insomnia on depression and anxiety: a systematic review and meta-analysis
Source: NPJ Digit Med. 2023 Mar 25;6:52. doi: 10.1038/s41746-023-00800-3 (PMC10039857; doi:10.1038/s41746-023-00800-3)
Supplement: Supplementary file 1 — Supplementary Materials [file 41746_2023_800_MOESM1_ESM.pdf]

Digital Cognitive Behavioral Therapy for Insomnia on Depression and Anxiety: A Systematic Review and Meta-analysis

Suonaa Lee[1,+], Jae Won Oh[2,+], Kyung Mee Park[1,2], San Lee[1,2*], Eun Lee[1,3*]

[1]Department of Psychiatry and the Institute of Behavioral Science in Medicine, Yonsei University College of Medicine, Seoul, Republic of Korea
[2]Department of Psychiatry, Yongin Severance Hospital, Yonsei University College of Medicine, Yongin, Republic of Korea
[3]Institute for Innovation in Digital Healthcare, Yonsei University, Seoul, Republic of Korea

[+]Co-first authors of the manuscript

* Corresponding authors:
San Lee, MD, MPH
Department of Psychiatry, Yongin Severance Hospital
363 Dongbaekjukjeondaero, Jung-dong, Giheung-gu, 16995
Yongin-si, Gyeonggi-do, South Korea
Tel.: +82-031-5189-8531; Fax: +82-031-5189-8565
E-mail: sanlee@yonsei.ac.kr

Eun Lee, M.D., Ph.D.
Department of Psychiatry, Yonsei University College of Medicine
50-1 Yonsei-ro, Seodaemun-gu, 03722 Seoul, South Korea
Telephone: +82 2 2228 1620; Fax: +82 2 313 0891
Email: leeeun@yuhs.ac

SUPPLEMENTARY MATERIALS

Table of Contents

Supplementary Table 1. Search Query in Intervention and Outcome Domains 3

Supplementary Figure 1. Risk of Bias Assessment 4

Supplementary Figure 2. Meta-analysis of the effect of dCBT-I on Sleep Diary Measures 5

Supplementary Figure 3. Meta-analysis of the effect of dCBT-I on Sleep Outcome (ISI only) 6

Supplementary Figure 4. The effect of dCBT-I on Depression Outcome (Comorbidity Removed) 7

Supplementary Figure 5. The effect of dCBT-I on Anxiety Outcome (Comorbidity Removed) 8

Supplementary Figure 6. The effect of dCBT-I on Sleep Outcome (Comorbidity Removed) 9

Supplementary Figure 7. The effect of fully automated dCBT-I 10

Supplementary Figure 8. The effect of dCBT-I on Sleep Outcome Funnel Plot 11

Supplementary Figure 9. The effect of dCBT-I on Depression Funnel Plot 12

Supplementary Figure 10. The effect of dCBT-I on Anxiety Funnel Plot 13

Supplementary Table 1. Search Query in Intervention and Outcome Domains

Intervention	Outcome
(CBT OR<br><br>cognitive behavioral therapy OR<br><br>cognitive therapy OR<br><br>behavioral therapy)<br><br>AND<br><br>(digital OR<br><br>internet OR<br><br>online OR<br><br>web OR<br><br>telephone OR<br><br>mobile OR<br><br>app OR<br><br>application OR<br><br>smartphone OR<br><br>computer OR<br><br>computerized OR<br><br>ehealth)	(depress* OR anxiety)<br><br>AND<br><br>(insomnia OR sleep)

Intention-to-treat	Study ID	Weight	D1	D2	D3	D4	D5	Overall
	Krieger 2019	1	+	+	+	+	+	+
	Kalmbach 2020	1	+	+	+	+	+	+
	Glozier 2018	1	+	!	+	+	-	-
	van der Zweerde 2019	1	-	+	+	!	+	-
	Espie 2018	1	+	+	+	!	+	!
	Christensen 2016	1	+	+	+	!	+	!
	Majd 2020	1	+	+	+	+	+	+
	Cheng 2019	1	+	+	-	+	+	!
	Sveen 2021	1	+	+	+	+	+	+
	van Straten 2014	1	+	+	+	!	+	!
	Lancee 2015	1	-	+	+	!	+	-
	Blom 2015	1	+	+	+	+	+	+
	Lancee 2016	1	+	!	+	!	+	!
	van der Zweerde 2020	1	+	+	+	!	+	!
	Lorenz 2019	1	+	!	+	!	+	!
	Bostock 2016	1	+	!	+	!	+	!
	Ahorsu 2020	1	+	+	+	+	+	+
	Pillai 2015	1	!	-	+	!	+	-
	Kyle 2020	1	+	+	+	!	+	!
	Freeman 2017	1	+	+	+	+	+	+
	Agyemang 2016	1	+	+	+	!	+	!
	Felder 2022	1	+	+	+	+	+	+

+ Low risk

! Some concerns

- High risk

D1 Randomisation process

D2 Deviations from the intended interventions

D3 Missing outcome data

D4 Measurement of the outcome

D5 Selection of the reported result

Supplementary Figure 1. Risk of Bias Assessment

Study Name	SMD	SE	Variance	Lower Limit	Upper Limit	z-value	p-value	Intervention	Control	Relative Weight
	Statistics for Each Study							Sample Size		
**Total Sleep Time**										
Agyemang et al. (2017)	1.236	0.412	0.170	0.427	2.044	2.996	0.003	14	14	5.90
Ahorsu et al. (2020)	0.063	0.112	0.013	-0.156	0.283	0.567	0.571	160	160	19.30
Blom et al. (2015)	-0.136	0.327	0.107	-0.776	0.504	-0.417	0.676	17	21	8.19
Lancee et al. (2015)	0.607	0.316	0.100	-0.012	1.227	1.922	0.055	25	18	8.55
Lancee et al. (2016)	0.053	0.261	0.068	-0.458	0.564	0.203	0.839	21	49	10.73
Pillai et al. (2015)	-0.069	0.434	0.188	-0.920	0.781	-0.160	0.873	13	9	5.47
van der Zweerde et al. (2019)	0.395	0.211	0.044	-0.018	0.807	1.874	0.061	45	47	13.23
van der Zweerde et al. (2020)	-0.003	0.195	0.038	-0.386	0.380	-0.014	0.989	55	50	14.09
van Straten et al. (2014)	0.571	0.188	0.035	0.203	0.939	3.039	0.002	59	59	14.53
**Overall random effects model**	0.263	0.116	0.013	0.036	0.490	2.268	0.023			
**Sleep Onset Latency**										
Agyemang et al. (2017)	-0.552	0.385	0.148	-1.307	0.203	-1.433	0.152	14	14	11.07
Ahorsu et al. (2020)	-0.210	0.112	0.013	-0.430	0.009	-1.875	0.061	160	160	14.48
Blom et al. (2015)	-0.396	0.329	0.109	-1.042	0.249	-1.204	0.229	17	21	11.89
Lancee et al. (2015)	-0.519	0.314	0.099	-1.135	0.097	-1.653	0.098	25	18	12.11
Pillai et al. (2015)	-0.713	0.447	0.200	-1.589	0.162	-1.597	0.110	13	9	10.16
van der Zweerde et al. (2019)	-0.508	0.212	0.045	-0.923	-0.092	-2.395	0.017	45	47	13.49
van der Zweerde et al. (2020)	-2.288	0.251	0.063	-2.780	-1.795	-9.107	0.000	55	50	12.99
van Straten et al. (2014)	-0.041	0.184	0.034	-0.402	0.320	-0.222	0.824	59	59	13.81
**Overall random effects model**	-0.645	0.253	0.064	-1.140	-0.150	-2.555	0.011			
**Wake After Sleep Onset**										
Agyemang et al. (2017)	-0.843	0.394	0.156	-1.616	-0.070	-2.138	0.033	14	14	19.42
Ahorsu et al. (2020)	-0.106	0.112	0.013	-0.325	0.114	-0.944	0.345	160	160	20.77
Lancee et al. (2015)	-0.901	0.324	0.105	-1.536	-0.266	-2.780	0.005	25	18	19.88
van der Zweerde et al. (2019)	-0.925	0.219	0.048	-1.356	-0.495	-4.217	0.000	45	47	20.42
van der Zweerde et al. (2020)	-4.745	0.381	0.145	-5.492	-3.998	-12.444	0.000	55	50	19.51
**Overall random effects model**	-1.480	0.655	0.429	-2.763	-0.196	-2.260	0.024			

Std Difference in Means and 95% CI

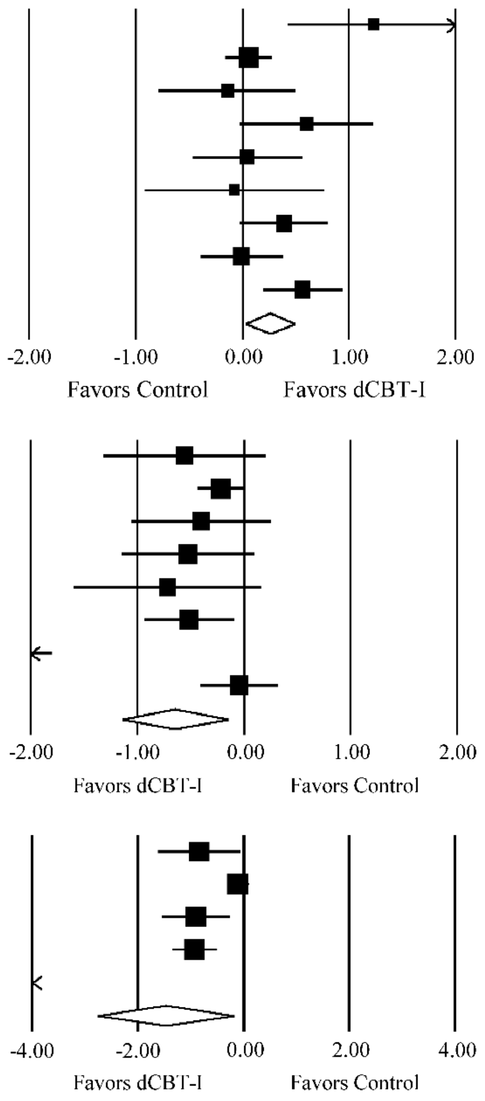

Supplementary Figure 2. Meta-analysis of the effect of dCBT-I on Sleep Diary Measures

Study Name	Statistics for Each Study							Sample Size		Relative Weight
	SMD	SE	Variance	Lower Limit	Upper Limit	z-value	p-value	Intervention	Control	
Agyemang et al. (2017)	-1.340	0.418	0.175	-2.159	-0.520	-3.203	0.001	14	14	2.58
Ahorsu et al. (2020)	-1.077	0.120	0.014	-1.312	-0.843	-9.003	0.000	160	160	7.08
Blom et al. (2015)	0.255	0.299	0.090	-0.332	0.842	0.851	0.395	22	23	3.89
Cheng et al. (2018)	-1.021	0.083	0.007	-1.184	-0.858	-12.271	0.000	358	300	7.70
Christensen et al. (2016)	-1.143	0.090	0.008	-1.320	-0.966	-12.650	0.000	248	333	7.59
Felder et al. (2020)	-0.799	0.153	0.023	-1.099	-0.500	-5.230	0.000	91	94	6.45
Freeman et al. (2017)	-0.711	0.049	0.002	-0.806	-0.615	-14.583	0.000	733	1142	8.13
Gloizer et al. (2019)	-0.395	0.234	0.055	-0.853	0.063	-1.690	0.091	40	35	4.92
Kalmbach et al. (2020)	-0.548	0.215	0.046	-0.969	-0.127	-2.551	0.011	46	44	5.26
Krieger et al. (2019)	-0.729	0.226	0.051	-1.171	-0.286	-3.228	0.001	34	54	5.06
Kyle et al. (2020)	-1.176	0.118	0.014	-1.408	-0.944	-9.927	0.000	155	181	7.10
Lancee et al. (2015)	-0.696	0.278	0.077	-1.241	-0.151	-2.502	0.012	36	22	4.20
Lancee et al. (2016)	0.145	0.238	0.057	-0.322	0.612	0.610	0.542	26	55	4.84
Lorenz et al. (2018)	-0.961	0.293	0.086	-1.536	-0.387	-3.279	0.001	25	27	3.98
Majd et al. (2020)	-0.759	0.117	0.014	-0.988	-0.529	-6.471	0.000	156	156	7.12
Pillai et al. (2015)	-0.936	0.456	0.208	-1.830	-0.043	-2.054	0.040	13	9	2.28
Sveen et al. (2021)	0.100	0.486	0.236	-0.853	1.053	0.206	0.837	9	8	2.07
van der Zweerde et al. (2019)	-1.533	0.237	0.056	-1.998	-1.068	-6.463	0.000	45	47	4.86
van der Zweerde et al. (2020)	-1.181	0.237	0.056	-1.644	-0.717	-4.991	0.000	43	41	4.87
**Overall random effects model**	-0.810	0.081	0.007	-0.968	-0.652	-10.033	0.000			

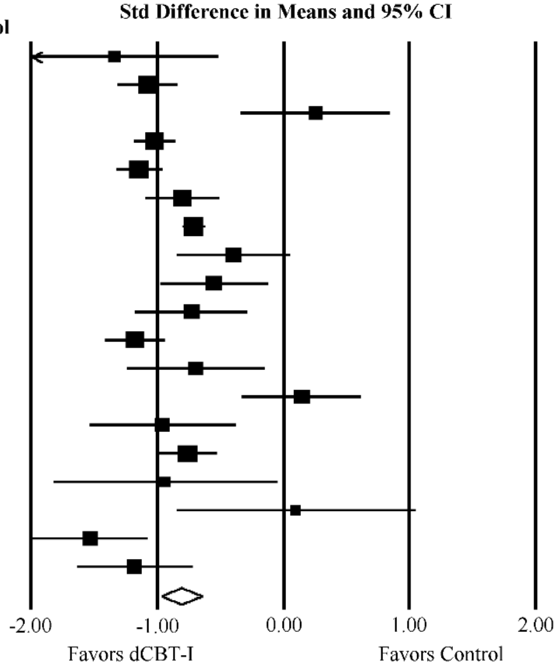

Supplementary Figure 3. Meta-analysis of the effect of dCBT-I on Sleep Outcome (ISI only)

Study Name	Statistics for Each Study							Sample Size		Relative Weight
	**SMD**	**SE**	**Variance**	**Lower Limit**	**Upper Limit**	**z-value**	**p-value**	**Intervention**	**Control**	
Blom et al. (2015)	-0.107	0.298	0.089	-0.692	0.478	-0.360	0.719	22	23	3.66
Bostock et al. (2016)	0.062	0.137	0.019	-0.207	0.331	0.452	0.651	98	116	6.46
Cheng et al. (2018)	-0.624	0.080	0.006	-0.781	-0.467	-7.786	0.000	358	300	7.47
Christensen et al. (2016)	-0.697	0.086	0.007	-0.866	-0.527	-8.068	0.000	248	333	7.38
Espie et al. (2019)	-0.019	0.064	0.004	-0.144	0.106	-0.292	0.770	468	517	7.70
Felder et al. (2020)	-0.386	0.148	0.022	-0.677	-0.095	-2.601	0.009	91	94	6.25
Freeman et al. (2017)	-0.435	0.048	0.002	-0.529	-0.341	-9.088	0.000	733	1142	7.88
Kalmbach et al. (2020)	-0.012	0.211	0.044	-0.426	0.401	-0.059	0.953	46	44	5.05
Krieger et al. (2019)	-0.432	0.222	0.049	-0.867	0.003	-1.945	0.052	34	53	4.85
Kyle et al. (2020)	-0.574	0.112	0.012	-0.792	-0.355	-5.137	0.000	155	181	6.94
Lancee et al. (2015)	-0.482	0.281	0.079	-1.033	0.068	-1.718	0.086	32	22	3.91
Lancee et al. (2016)	0.023	0.238	0.057	-0.444	0.489	0.095	0.924	26	55	4.58
Lorenz et al. (2018)	-0.431	0.281	0.079	-0.982	0.119	-1.536	0.124	25	27	3.91
Majd et al. (2020)	-0.943	0.119	0.014	-1.177	-0.709	-7.901	0.000	156	156	6.80
Sveen et al. (2021)	-0.166	0.501	0.251	-1.147	0.816	-0.330	0.741	8	8	1.83
van der Zweerde et al. (2018)	-0.839	0.218	0.047	-1.266	-0.413	-3.857	0.000	45	47	4.93
Van der Zweerde et al. (2020)	-0.378	0.220	0.048	-0.809	0.054	-1.716	0.086	43	41	4.88
Van Straten et al. (2014)	-0.444	0.186	0.035	-0.809	-0.079	-2.382	0.017	59	59	5.51
**Overall random effects model**	-0.405	0.077	0.006	-0.556	-0.254	-5.264	0.000			

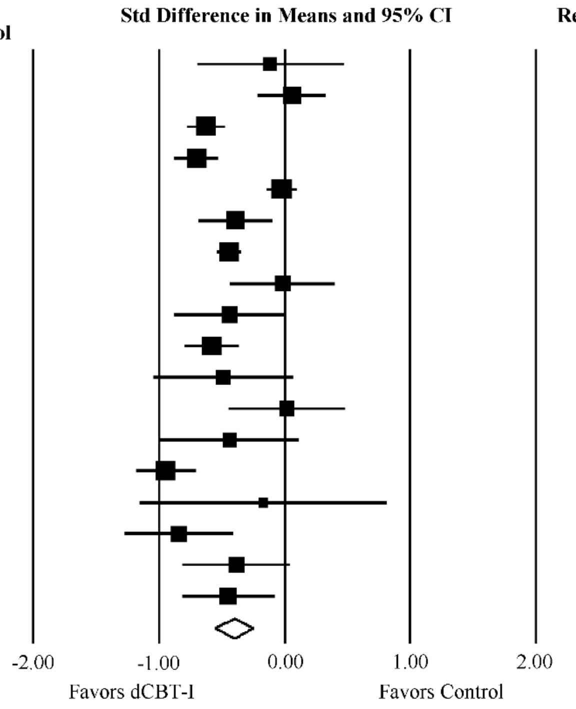

Supplementary Figure 4. The effect of dCBT-I on Depression Outcome (Comorbidity Removed)

Study Name	Statistics for Each Study							Sample Size		Relative Weight
	SMD	SE	Variance	Lower Limit	Upper Limit	z-value	p-value	Intervention	Control	
Bostock et al. (2016)	-0.141	0.137	0.019	-0.410	0.128	-1.025	0.305	98	116	7.94
Christensen et al. (2016)	-0.522	0.085	0.007	-0.690	-0.355	-6.127	0.000	248	333	11.29
Espie et al. (2019)	-0.014	0.064	0.004	-0.139	0.111	-0.224	0.823	468	517	12.78
Felder et al. (2020)	-0.405	0.149	0.022	-0.696	-0.114	-2.724	0.006	91	94	7.34
Freeman et al. (2017)	-0.313	0.048	0.002	-0.407	-0.220	-6.579	0.000	733	1142	13.80
Kyle et al. (2020)	-0.346	0.110	0.012	-0.562	-0.129	-3.134	0.002	155	181	9.59
Lancee et al. (2015)	-0.393	0.280	0.078	-0.941	0.155	-1.405	0.160	32	22	3.16
Lancee et al. (2016)	-0.191	0.238	0.057	-0.659	0.276	-0.803	0.422	26	55	4.03
Lorenz et al. (2018)	-0.320	0.279	0.078	-0.868	0.227	-1.147	0.252	25	27	3.16
Majd et al. (2020)	-0.311	0.114	0.013	-0.534	-0.088	-2.730	0.006	156	156	9.35
Pillai et al. (2015)	-0.210	0.435	0.189	-1.062	0.642	-0.482	0.630	13	9	1.48
Sveen et al. (2021)	0.215	0.501	0.251	-0.768	1.198	0.429	0.668	8	8	1.14
van der Zweerde et al. (2018)	-0.521	0.212	0.045	-0.936	-0.105	-2.456	0.014	45	47	4.76
Van der Zweerde et al. (2020)	-0.053	0.218	0.048	-0.481	0.375	-0.243	0.808	43	41	4.57
Van Straten et al. (2014)	-0.526	0.187	0.035	-0.893	-0.159	-2.810	0.005	59	59	5.61
**Overall random effects model**	-0.295	0.056	0.003	-0.404	-0.186	-5.291	0.000			

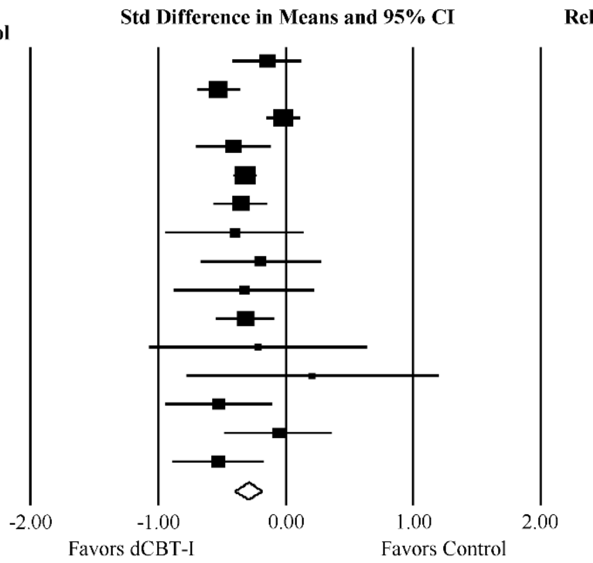

Supplementary Figure 5. The effect of dCBT-I on Anxiety Outcome (Comorbidity Removed)

Study Name	Statistics for Each Study							Sample Size		Relative Weight
	SMD	SE	Variance	Lower Limit	Upper Limit	z-value	p-value	Intervention	Control	
Blom et al. (2015)	0.255	0.299	0.090	-0.332	0.842	0.851	0.395	22	23	4.37
Bostock et al. (2016)	-0.749	0.142	0.020	-1.027	-0.471	-5.279	0.000	98	116	5.96
Cheng et al. (2018)	-1.021	0.083	0.007	-1.184	-0.858	-12.271	0.000	358	300	6.40
Christensen et al. (2016)	-1.143	0.090	0.008	-1.320	-0.966	-12.650	0.000	248	333	6.36
Espie et al. (2019)	-0.041	0.064	0.004	-0.166	0.084	-0.646	0.518	468	517	6.50
Felder et al. (2020)	-0.799	0.153	0.023	-1.099	-0.500	-5.230	0.000	91	94	5.86
Freeman et al. (2017)	-0.711	0.049	0.002	-0.806	-0.615	-14.583	0.000	733	1142	6.57
Kalmbach et al. (2020)	-0.548	0.215	0.046	-0.969	-0.127	-2.551	0.011	46	44	5.25
Krieger et al. (2019)	-0.729	0.226	0.051	-1.171	-0.286	-3.228	0.001	34	54	5.13
Kyle et al. (2020)	-1.176	0.118	0.014	-1.408	-0.944	-9.927	0.000	155	181	6.15
Lancee et al. (2015)	-0.696	0.278	0.077	-1.241	-0.151	-2.502	0.012	36	22	4.59
Lancee et al. (2016)	0.145	0.238	0.057	-0.322	0.612	0.610	0.542	26	55	5.00
Lorenz et al. (2018)	-0.961	0.293	0.086	-1.536	-0.387	-3.279	0.001	25	27	4.43
Majd et al. (2020)	-0.759	0.117	0.014	-0.988	-0.529	-6.471	0.000	156	156	6.16
Pillai et al. (2015)	-0.936	0.456	0.208	-1.830	-0.043	-2.054	0.040	13	9	3.01
Sveen et al. (2021)	0.100	0.486	0.236	-0.853	1.053	0.206	0.837	9	8	2.80
van der Zweerde et al. (2018)	-1.533	0.237	0.056	-1.998	-1.068	-6.463	0.000	45	47	5.01
van der Zweerde et al. (2020)	-1.181	0.237	0.056	-1.644	-0.717	-4.991	0.000	43	41	5.02
van Straten et al. (2014)	-1.059	0.197	0.039	-1.444	-0.673	-5.385	0.000	59	59	5.43
**Overall random effects model**	-0.739	0.107	0.011	-0.948	-0.530	-6.924	0.000			

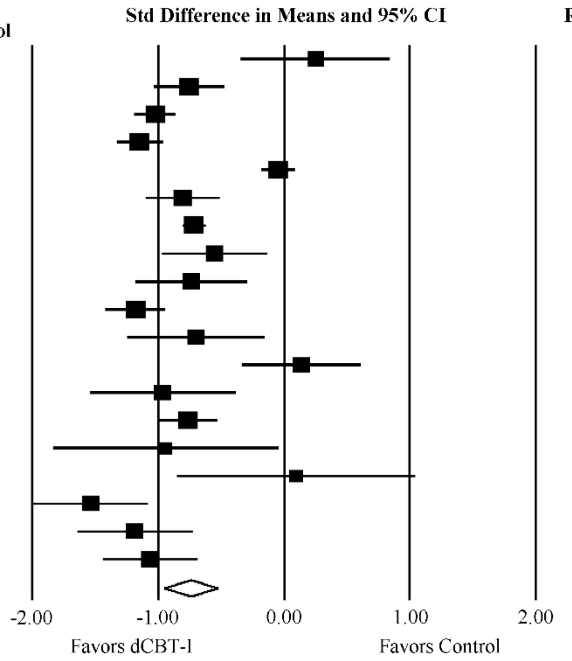

Supplementary Figure 6. The effect of dCBT-I on Sleep Outcome (Comorbidity Removed)

Study Name	SMD	SE	Variance	Lower Limit	Upper Limit	z-value	p-value	Intervention	Control	Relative Weight
	Statistics for Each Study							Sample Size		
**Depression**										
Agyemang et al. (2017)	-0.463	0.383	0.147	-1.213	0.288	-1.208	0.227	14	14	3.51
Ahorsu et al. (2020)	-0.719	0.115	0.013	-0.945	-0.493	-6.232	0.000	160	160	8.55
Bostock et al. (2016)	0.062	0.137	0.019	-0.207	0.331	0.452	0.651	98	116	8.07
Cheng et al. (2018)	-0.624	0.080	0.006	-0.781	-0.467	-7.786	0.000	358	300	9.24
Christensen et al. (2016)	-0.697	0.086	0.007	-0.866	-0.527	-8.068	0.000	248	333	9.13
Espie et al. (2019)	-0.019	0.064	0.004	-0.144	0.106	-0.292	0.770	468	517	9.50
Felder et al. (2020)	-0.386	0.148	0.022	-0.677	-0.095	-2.601	0.009	91	94	7.82
Freeman et al. (2017)	-0.435	0.048	0.002	-0.529	-0.341	-9.088	0.000	733	1142	9.71
Glozier et al. (2019)	-0.202	0.232	0.054	-0.656	0.253	-0.869	0.385	40	35	5.95
Kalmbach et al. (2020)	-0.012	0.211	0.044	-0.426	0.401	-0.059	0.953	46	44	6.40
Kyle et al. (2020)	-0.574	0.112	0.012	-0.792	-0.355	-5.137	0.000	155	181	8.63
Lorenz et al. (2018)	-0.431	0.281	0.079	-0.982	0.119	-1.536	0.124	25	27	5.01
Majd et al. (2020)	-0.943	0.119	0.014	-1.177	-0.709	-7.901	0.000	156	156	8.47
**Overall random effects model**	-0.432	0.089	0.008	-0.606	-0.257	-4.851	0.000			
**Anxiety**										
Agyemang et al. (2017)	0.114	0.378	0.143	-0.627	0.856	0.302	0.763	14	14	2.28
Ahorsu et al. (2020)	-0.513	0.114	0.013	-0.735	-0.290	-4.512	0.000	160	160	10.14
Bostock et al. (2016)	-0.141	0.137	0.019	-0.410	0.128	-1.025	0.305	98	116	8.76
Christensen et al. (2016)	-0.522	0.085	0.007	-0.690	-0.355	-6.127	0.000	248	333	11.92
Espie et al. (2019)	-0.014	0.064	0.004	-0.139	0.111	-0.224	0.823	468	517	13.23
Felder et al. (2020)	-0.405	0.149	0.022	-0.696	-0.114	-2.724	0.006	91	94	8.16
Freeman et al. (2017)	-0.313	0.048	0.002	-0.407	-0.220	-6.579	0.000	733	1142	14.11
Gloizer et al. (2019)	-0.019	0.215	0.046	-0.439	0.402	-0.088	0.930	45	42	5.40
Kyle et al. (2020)	-0.346	0.110	0.012	-0.562	-0.129	-3.134	0.002	155	181	10.35
Lorenz et al. (2018)	-0.320	0.279	0.078	-0.868	0.227	-1.147	0.252	25	27	3.72
Majd et al. (2020)	-0.311	0.114	0.013	-0.534	-0.088	-2.730	0.006	156	156	10.12
Pillai et al. (2015)	-0.210	0.435	0.189	-1.062	0.642	-0.482	0.630	13	9	1.79
**Overall random effects model**	-0.287	0.062	0.004	-0.408	-0.166	-4.636	0.000			
**Sleep Outcome**										
Agyemang et al. (2017)	-1.340	0.418	0.175	-2.159	-0.520	-3.203	0.001	14	14	4.09
Ahorsu et al. (2020)	-1.077	0.120	0.014	-1.312	-0.843	-9.003	0.000	160	160	8.02
Bostock et al. (2016)	-0.749	0.142	0.020	-1.027	-0.471	-5.279	0.000	98	116	7.75
Cheng et al. (2018)	-1.021	0.083	0.007	-1.184	-0.858	-12.271	0.000	358	300	8.39
Christensen et al. (2016)	-1.143	0.090	0.008	-1.320	-0.966	-12.650	0.000	248	333	8.33
Espie et al. (2019)	-0.041	0.064	0.004	-0.166	0.084	-0.646	0.518	468	517	8.54
Felder et al. (2020)	-0.799	0.153	0.023	-1.099	-0.500	-5.230	0.000	91	94	7.61
Freeman et al. (2017)	-0.711	0.049	0.002	-0.806	-0.615	-14.583	0.000	733	1142	8.64
Gloizer et al. (2019)	-0.395	0.234	0.055	-0.853	0.063	-1.690	0.091	40	35	6.46
Kalmbach et al. (2020)	-0.548	0.215	0.046	-0.969	-0.127	-2.551	0.011	46	44	6.74
Kyle et al. (2020)	-1.176	0.118	0.014	-1.408	-0.944	-9.927	0.000	155	181	8.03
Lorenz et al. (2018)	-0.961	0.293	0.086	-1.536	-0.387	-3.279	0.001	25	27	5.62
Majd et al. (2020)	-0.759	0.117	0.014	-0.988	-0.529	-6.471	0.000	156	156	8.05
Pillai et al. (2015)	-0.936	0.456	0.208	-1.830	-0.043	-2.054	0.040	13	9	3.72
**Overall random effects model**	-0.813	0.116	0.013	-1.040	-0.586	-7.012	0.000			

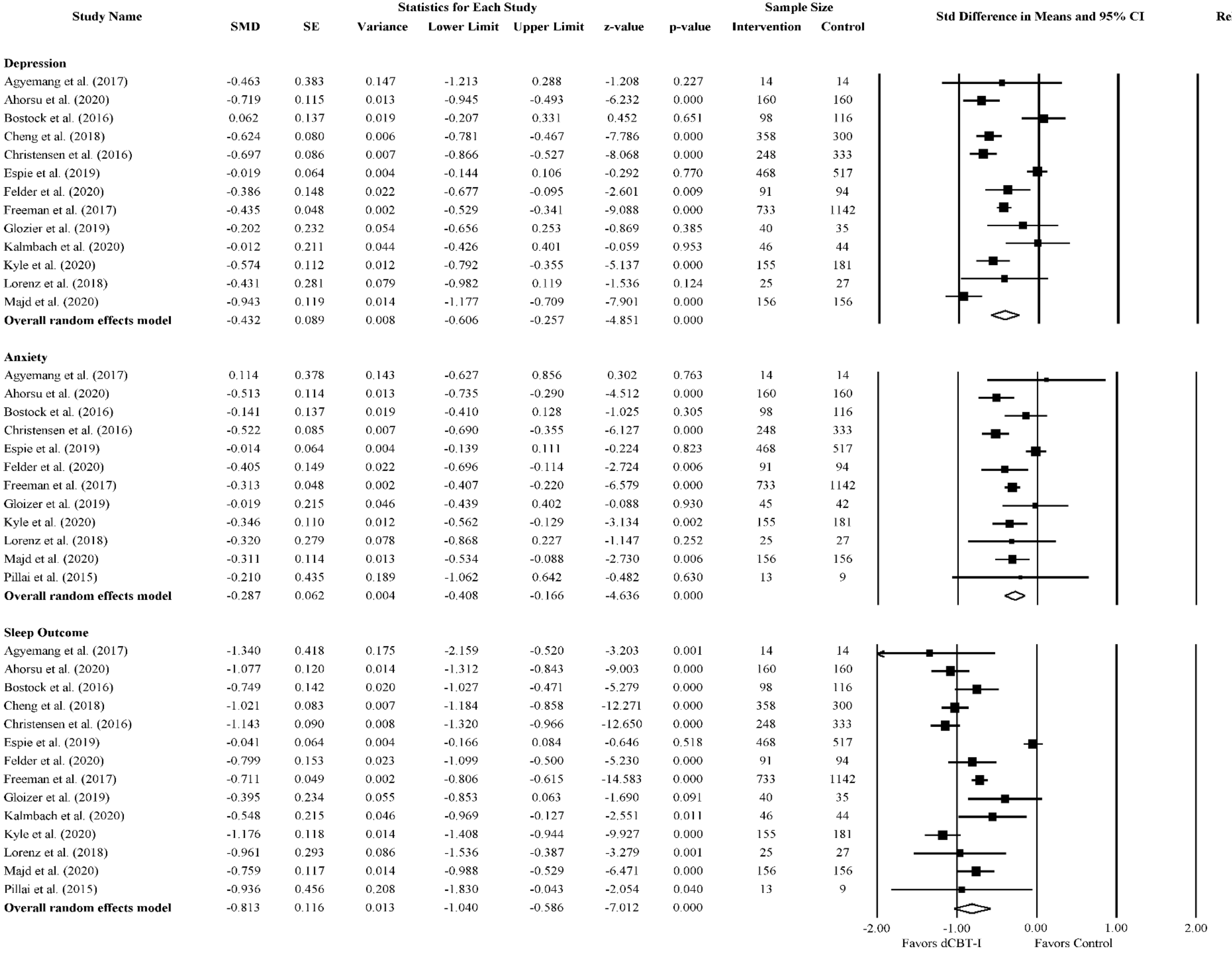

Supplementary Figure 7. The effect of fully automated dCBT-I

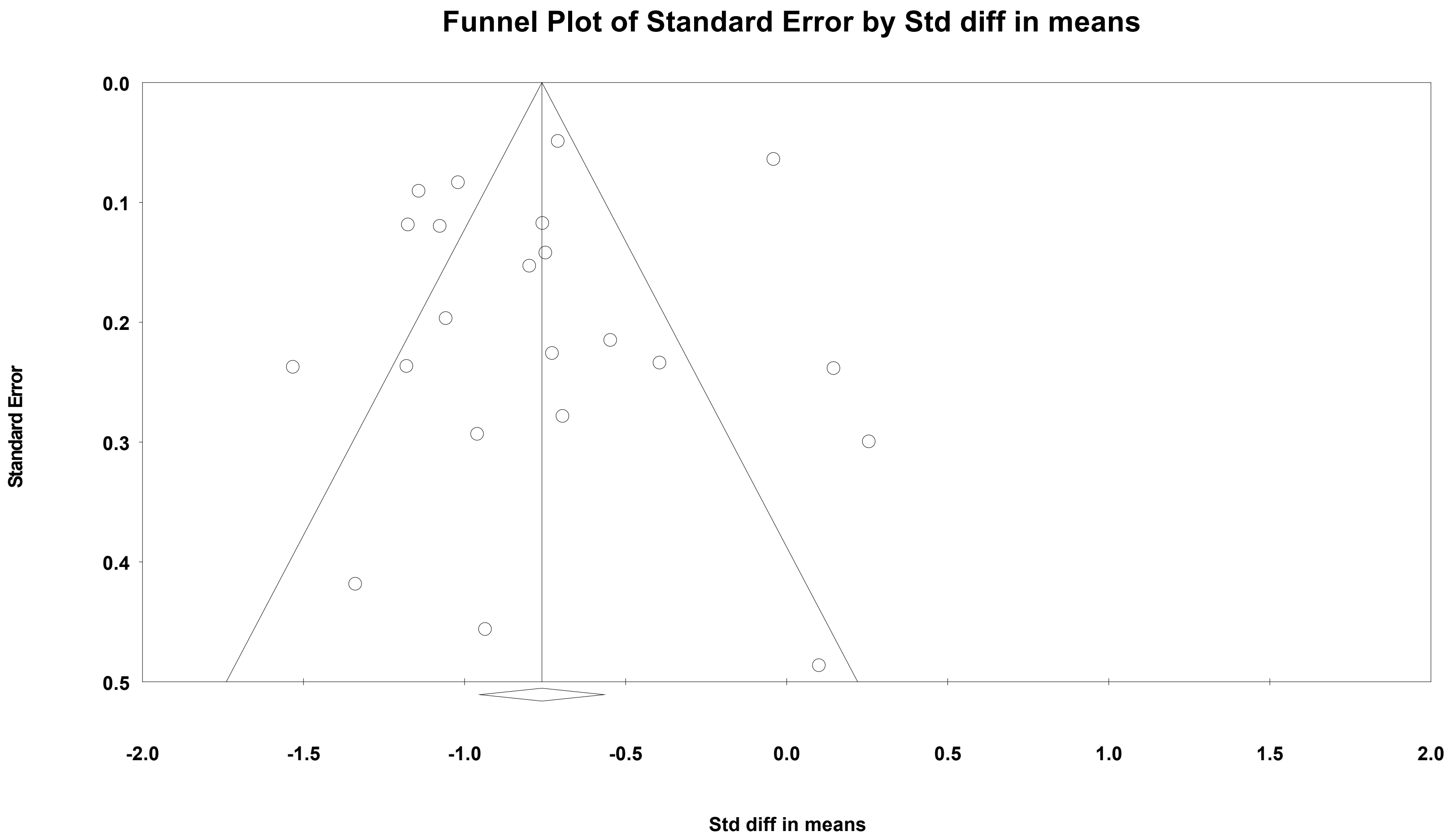

Supplementary Figure 8. The effect of dCBT-I on Sleep Outcome Funnel Plot

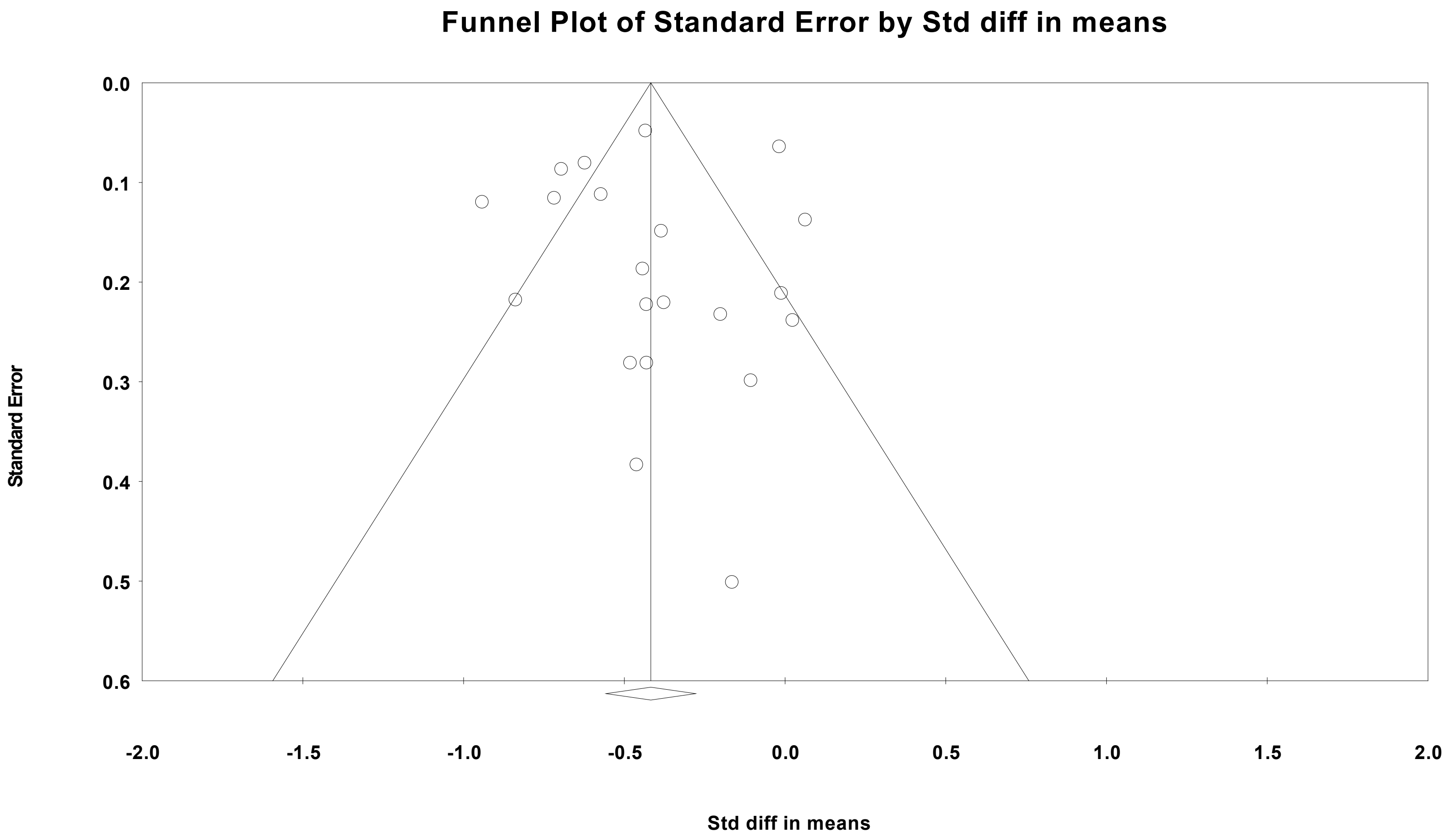

Supplementary Figure 9. The effect of dCBT-I on Depression Funnel Plot

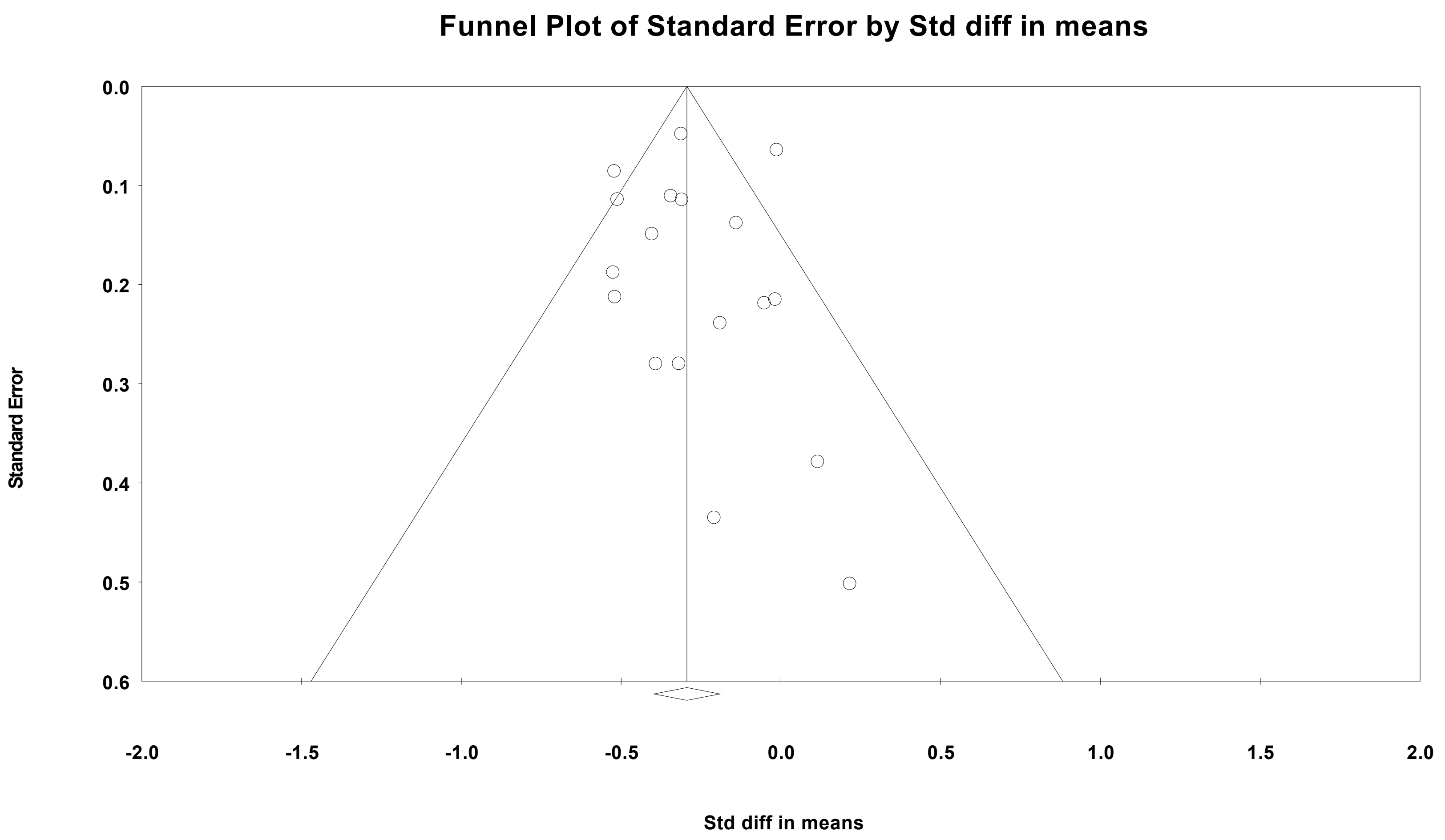

Supplementary Figure 10. The effect of dCBT-I on Anxiety Funnel Plot
