# Supplementary material for: Digital cognitive behavioral therapy for insomnia on depression and anxiety: a systematic review and meta-analysis
Source: NPJ Digit Med. 2023 Mar 25;6:52. doi: 10.1038/s41746-023-00800-3 (PMC10039857; doi:10.1038/s41746-023-00800-3)
Supplement: Supplementary file 2 — REPORTING SUMMARY [file 41746_2023_800_MOESM2_ESM.pdf]

nature portfolio

Corresponding author(s): San Lee and Eun Lee

Last updated by author(s): Feb 6, 2023

Reporting Summary

Nature Portfolio wishes to improve the reproducibility of the work that we publish. This form provides structure for consistency and transparency in reporting. For further information on Nature Portfolio policies, see our Editorial Policies and the Editorial Policy Checklist.

Statistics

For all statistical analyses, confirm that the following items are present in the figure legend, table legend, main text, or Methods section.

n/a	Confirmed	
☐	☒	The exact sample size (*n*) for each experimental group/condition, given as a discrete number and unit of measurement
☐	☒	A statement on whether measurements were taken from distinct samples or whether the same sample was measured repeatedly
☐	☒	The statistical test(s) used AND whether they are one- or two-sided<br>*Only common tests should be described solely by name; describe more complex techniques in the Methods section.*
☐	☒	A description of all covariates tested
☐	☒	A description of any assumptions or corrections, such as tests of normality and adjustment for multiple comparisons
☐	☒	A full description of the statistical parameters including central tendency (e.g. means) or other basic estimates (e.g. regression coefficient) AND variation (e.g. standard deviation) or associated estimates of uncertainty (e.g. confidence intervals)
☒	☐	For null hypothesis testing, the test statistic (e.g. *F*, *t*, *r*) with confidence intervals, effect sizes, degrees of freedom and *P* value noted<br>*Give P values as exact values whenever suitable.*
☒	☐	For Bayesian analysis, information on the choice of priors and Markov chain Monte Carlo settings
☐	☒	For hierarchical and complex designs, identification of the appropriate level for tests and full reporting of outcomes
☐	☒	Estimates of effect sizes (e.g. Cohen's *d*, Pearson's *r*), indicating how they were calculated

Our web collection on statistics for biologists contains articles on many of the points above.

Software and code

Policy information about availability of computer code

Data collection	No computer code/algorithms used
Data analysis	No computer code/algorithms used

For manuscripts utilizing custom algorithms or software that are central to the research but not yet described in published literature, software must be made available to editors and reviewers. We strongly encourage code deposition in a community repository (e.g. GitHub). See the Nature Portfolio guidelines for submitting code & software for further information.

Data

Policy information about availability of data

All manuscripts must include a data availability statement. This statement should provide the following information, where applicable:

- Accession codes, unique identifiers, or web links for publicly available datasets
- A description of any restrictions on data availability
- For clinical datasets or third party data, please ensure that the statement adheres to our policy

Data collected and used in this meta-analysis can be requested from the corresponding authors.

Human research participants

Policy information about studies involving human research participants and Sex and Gender in Research.

Reporting on sex and gender	The current research was a systemic review and meta-analysis, hence no specific reporting of the sex and gender from individual studies were necessary.
Population characteristics	Any studies that met the selection criteria listed below were included. Participants had to be adult patients aged ≥18 years; have been formally diagnosed or had self-reported symptoms of insomnia; with reported measures of depressive or anxiety symptoms; involving either a dCBT-I intervention delivered by digital technology including computer, Internet and smartphone applications, or a control group with other interventions for managing insomnia, active controls, waitlist, or participants who underwent usual care.
Recruitment	Studies were searched and collected through PubMed, PsycINFO, Embase and Cochrane
Ethics oversight	All ethical approvals were waived as the current study is a systematic review and meta-analysis using literature searched from above mentioned databases.

Note that full information on the approval of the study protocol must also be provided in the manuscript.

Field-specific reporting

Please select the one below that is the best fit for your research. If you are not sure, read the appropriate sections before making your selection.

☒ Life sciences ☐ Behavioural & social sciences ☐ Ecological, evolutionary & environmental sciences

For a reference copy of the document with all sections, see nature.com/documents/nr-reporting-summary-flat.pdf

Life sciences study design

All studies must disclose on these points even when the disclosure is negative.

Sample size	Sample size calculation not necessary for systematic review/meta-analysis
Data exclusions	Strict inclusion criteria developed in including the relevant articles: 1) comprised adult patients aged ≥18 years; 2) have been formally diagnosed or had self-reported symptoms of insomnia defined by any edition of the DSM, International Classification of Sleep Disorders, or International Classification of Diseases; 3) with reported measures of depressive or anxiety symptoms; 4) involving a dCBT-I intervention delivered by digital technology including computer, Internet and smartphone applications, alongside a control group with other interventions for managing insomnia, active controls, waitlist, or participants who underwent usual care; 5) adopted an RCT design.
Replication	Replication can be done using the search terms provided. Specific dates for the search period has also been addressed.
Randomization	N/A for systematic review/meta-analysis, only RCTs were included in the current study.
Blinding	N/A for systematic review/meta-analysis.

Reporting for specific materials, systems and methods

We require information from authors about some types of materials, experimental systems and methods used in many studies. Here, indicate whether each material, system or method listed is relevant to your study. If you are not sure if a list item applies to your research, read the appropriate section before selecting a response.

Materials & experimental systems

n/a	Involved in the study	
☒	☐	Antibodies
☒	☐	Eukaryotic cell lines
☒	☐	Palaeontology and archaeology
☒	☐	Animals and other organisms
☒	☐	Clinical data
☒	☐	Dual use research of concern

Methods

n/a	Involved in the study	
☒	☐	ChIP-seq
☒	☐	Flow cytometry
☒	☐	MRI-based neuroimaging
